# Peripheral Serotonergic Activation in Severe Aortic Stenosis: A Biochemical Perspective

**DOI:** 10.3390/ijms262110250

**Published:** 2025-10-22

**Authors:** Denisa Bianca Mercean, Raluca Tomoaia, Ioana Berindan-Neagoe, Liviuţa Budişan, Dana Pop, Adela Mihaela Șerban, Carmen Stanca Melincovici, Carmen Mihaela Mihu

**Affiliations:** 1 1st Department of Morpho-functional Sciences, “Iuliu Haţieganu” University of Medicine and Pharmacy, 400012 Cluj-Napoca, Romania; melincovici@umfcluj.ro (C.S.M.); carmenmihu@umfcluj.ro (C.M.M.); 2 Cardiology Department, Heart Institute “N. Stăncioiu”, 400001 Cluj-Napoca, Romania; adelamserban@yahoo.com; 3 4th Department of Internal Medicine, “Iuliu Haţieganu” University of Medicine and Pharmacy, 400012 Cluj-Napoca, Romania; raluca.tomoaia@gmail.com (R.T.); pop67dana@gmail.com (D.P.); 4 Cardiology Department, Rehabilitation Hospital, 400347 Cluj-Napoca, Romania; 5 Genomics Department, MEDFUTURE Institute of Biomedical Research, “Iuliu Hatieganu” University of Medicine and Pharmacy, 23 Marinescu Street, 400337 Cluj-Napoca, Romania; ioananeagoe29@gmail.com (I.B.-N.); lbudisan@yahoo.com (L.B.); 6 Doctoral School, “Iuliu Haţieganu” University of Medicine and Pharmacy, 8 Victor Babeş Street, 400012 Cluj-Napoca, Romania; 7 Academy of Medical Sciences, 1 Ion C. Brătianu Boulevard, 3rd Sector, 030167 Bucharest, Romania; 8 Radiology and Imaging Department, County Emergency Hospital, 400006 Cluj-Napoca, Romania

**Keywords:** aortic stenosis, serotonin, 5-HT, 5-HIAA, ELISA

## Abstract

The involvement of the serotoninergic system in the pathogenesis of calcific aortic stenosis introduced a novel dimension to our understanding of this complex cardiovascular condition. This study aimed to assess serotonin (5-HT) and its main metabolite, 5-Hydroxyindoleacetic acid (5-HIAA) in patients with severe aortic valve stenosis (AS). The study employed a case–control design, including 76 patients who underwent transthoracic echocardiography, computed tomography (CT), and peripheral blood sampling. Serum concentrations of 5-HT and 5-HIAA were quantified using enzyme-linked immunosorbent assay (ELISA). The severe aortic valve stenosis group exhibited significantly elevated levels of 5-HT and 5-HIAA compared to the control group (5-HT 1066.5 ng/mL (IQR = 961.9–1112 ng/mL) vs. 977.4 ng/mL (IQR = 394.3–1097.9 ng/mL); *p* = 0.034 and 5-HIAA 57 ± 12.7 ng/mL vs. 47.5 ± 15.3 ng/mL; *p* = 0.004, respectively). Receiver operating characteristic (ROC) analysis revealed that 5-HT predicted severe AS with a sensitivity of 73.7% and specificity of 50% at a cut-off level > 973.5 ng/mL, whereas 5-HIAA exhibited a sensitivity of 86.8% and specificity of 47.4% when a cut-off level > 45.49 ng/mL was used. This study showed a significant elevation in the 5-HT and 5-HIAA among patients with severe AS, further supporting the potential involvement of the peripheral serotonergic system in the pathophysiology of this condition.

## 1. Introduction

Calcific aortic stenosis (AS) ranks as the most prevalent valvular heart disease in developed countries [1]. It involves the gradual alteration and thickening of the aortic valve leaflets attributed to fibro-calcific transformations. While calcific AS was traditionally perceived as a passive degenerative process resulting from prolonged mechanical stress, recent insights highlight its multifaceted nature as an outcome of intricately regulated and complex cellular mechanisms extending beyond traditional paradigms [2].

In 2008, Liberman et al. [3] followed by Miller et al. [4] were the first to show the elevated oxidative stress in calcified areas of stenotic aortic valves. A few months later, the latter research team hypothesized that oxidative stress may be found in heart valves exposed to high concentrations of serotonin. They reported two key findings: high concentrations of serotonin or dopamine were shown to increase in superoxide radicals in human heart valves and vascular tissue, and monoamine oxidase-A (MAO-A) emerged as a novel source of superoxide in human heart valves [5].

Serotonin (5-hydroxytryptamine, 5-HT) is a multifunctional biogenic amine acting both as a neurotransmitter and as a peripheral signaling molecule. It regulates numerous systemic functions, including mood, sleep, memory, gastrointestinal motility, and pulmonary activity, and exerts important effects in the cardiovascular system, where it modulates vasomotor tone, myocardial contractility, heart rate, and fibroblast function through receptor-specific mechanisms [6].

Valvular heart disease associated with increased serotonin levels has been observed with carcinoid tumor [7] and after prolonged administration of the diet drug combination of fenfluramine–phentermine [8]. Moreover, rats subjected to prolonged serotonin administration exhibit heightened cell proliferation and thickening of the heart valves, mirroring the alterations reported in individuals with carcinoid heart disease [9,10]. These changes are more frequent in the tricuspid and pulmonary valves than in the left-side valves, presumably because lungs are a major site of metabolism for serotonin and other amines [7], and the aortic and mitral valves therefore are exposed to lower concentrations of serotonin. SERAOPI [11] stands as the pioneering and sole study thus far to examine serotonin levels in severe aortic stenosis. Notably, it revealed, for the first time, elevated levels of arterial plasma 5-HT, its metabolite 5-HIAA, and increased platelet activation in individuals with severe AS. These findings imply a potential involvement of the peripheral serotoninergic system in the processes of cardiac remodeling and valve dysfunction associated with AS.

Considering the current absence of pharmaceutical interventions to decelerate the progression of aortic stenosis and the sole recourse of valve replacement for treating this valvular heart disease, there arises a pressing demand for novel therapeutic targets. Hence, our objective is to bring new evidence that incriminates the serotoninergic system in the physiopathology of severe aortic stenosis (AS), with the underlying hypothesis that individuals afflicted with severe aortic stenosis exhibit elevated levels of serum serotonin and its principal metabolite, 5-HIAA, in comparison to a matched control group. The findings of our study aim to provide a foundation for future research, with the serotoninergic system emerging as a viable therapeutic target for further exploration.

## 2. Results

The total cohort included a number of 76 patients, sorted into two equal groups of 38 each, based on the presence or absence of a diagnosis of severe aortic stenosis.

Table 1 summarizes the patients’ demographic characteristics, comorbid conditions, cardiovascular medication, and echocardiographic measurements of the patients according to the presence of AS, and Table 2 presents the laboratory parameters of the two groups. A total of 37 patients (48.7%) were male, and the mean age of the study population was 76.5 ± 5 years. Concerning demographic characteristics, there were no discernible differences between the two groups. The most common chronic medical condition in the study cohort was heart failure (93.4%), followed by arterial hypertension (90.7%). Atrial fibrillation was higher in patients from the control group (*p* = 0.015). No other comorbid condition showed significant differences between groups. In terms of the primary cardiovascular medication taken by patients, the only distinction was noted in the class of angiotensin–aldosterone system inhibitors, showing a significantly higher consumption among individuals in the control group (*p* = 0.0008).

**Figure 1:**
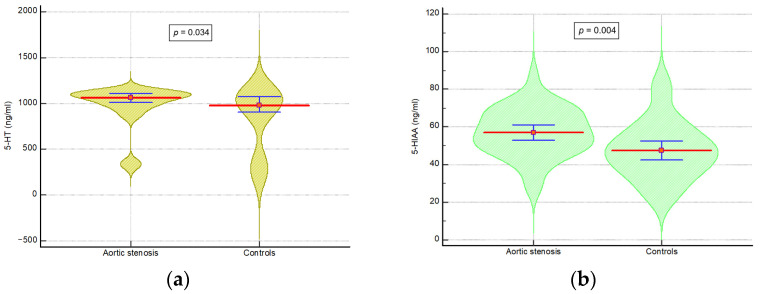
Comparison of median 5-HT levels (**a**) and average 5-HIAA levels (**b**) between the two groups. 5-HT: 5-Hydroxytryptamine; 5-HIAA: 5-Hydroxyindolacetic acid. The red line represents the mean value, and the blue line indicates the 95% confidence interval (CI) of the mean for each group.

Regarding routine laboratory parameters, patients from the control group exhibited lower NT-proBNP levels (*p* = 0.0001). There were no statistically significant differences noted in hemoglobin, creatinine, lipid profile, or serum glucose levels. The median serotonin/5-HT level was 1036.4 ng/mL with an interquartile range of 903.4 to 1106.6 ng/mL. The mean 5-HIAA level was 52.3 ng/mL with an SD of 14.7 ng/mL. The levels of 5-HT and 5-HIAA were found to be higher in the case group when compared to the control group (*p* = 0.034 and *p* = 0.004, respectively) (Figure 1).

In terms of echocardiographic parameters, patients with severe AS had greater interventricular septum and posterior wall thickness, but lower left ventricular filling pressures (E/e’) (*p* < 0.0001). In individuals with severe aortic stenosis (case group), additional echocardiographic parameters were assessed, including the Doppler velocity index (0.195 ± 0.05), aortic valve area (0.58 cm^2^ ± 0.22), and the mean gradient across the aortic valve, exhibiting a median value of 52 mmHg (IQR = 45–66 mmHg). Moreover, the AS group underwent computed tomography scans to evaluate the aortic valve calcium score, revealing a median value of 2881.5 Agatston units (IQR = 1871–5267 Agatston units).

**Figure 2:**
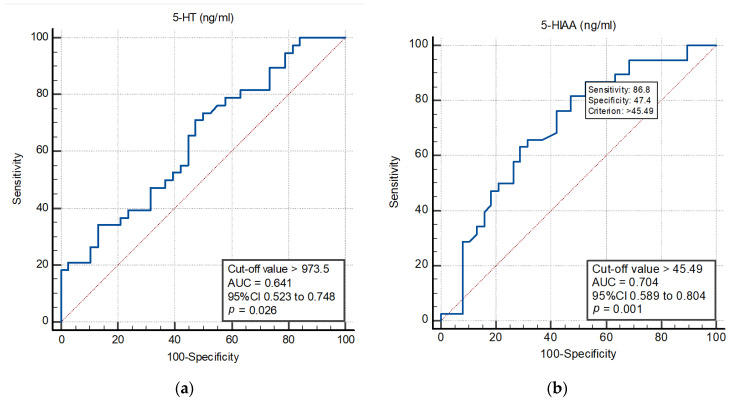
Receiver operating characteristic (ROC) curve analysis of 5-HT (**a**) and 5-HIAA (**b**) to predict the severe aortic stenosis diagnosis. AUC: area under curve; 5-HT: 5-Hydroxytryptamine; 5-HIAA: 5-Hydroxy indolacetic acid.

The ROC curve analysis explored the discriminatory capability of the 5-HT (Figure 2a) and 5-HIAA (Figure 2b) for the severe AS diagnosis. For 5-HT, the area under the curve (AUC) was 0.641 (95% CI: 0.523–0.748; *p* = 0.026). Using a cut-off level > 973.5 ng/mL, the 5-HT predicted the severe AS diagnosis with a sensitivity of 73.7% and specificity of 50% (Figure 2a). For 5-HIAA, the AUC was 0.704 (95% CI: 0.589–0.804; *p* = 0.001). A post hoc power analysis showed that with 38 participants per group, the study had sufficient power to detect significant differences in the AUC for both 5-HT (AUC 0.641, min 30 patients needed) and 5-HIAA (AUC 0.704, minimum 25 patients needed). In predicting the severe AS diagnosis, the 5-HIAA exhibited a sensitivity of 86.8% and specificity of 47.4% when a cut-off level > 45.49 ng/mL was used (Figure 2b).

In the AS group, Spearman’s rank correlation coefficients were calculated (Table 3). The serotonin level demonstrated a weak positive correlation with the following: aortic valve maximum velocity (r = 0.23), aortic valve mean pressure gradient (r = 0.2) and left ventricular posterior wall thickness (r = 0.22), NT-proBNP (r = 0.27), and a weak negative correlation with age (r = −0.31). As for 5-HIAA, a weak positive correlation with Vmax (r = 0.26), interventricular septum (r = 0.31), LVPW (r = 0.2), E/e’ (r = 0.22), and NT-proBNP (r = 0.2) was proved. Neither showed correlation with the aortic valve calcium score.

## 3. Discussion

The present research provides new evidence of increased levels of serum 5-HT, along with its MAO-A-dependent metabolite, 5-HIAA, in individuals with severe AS.

Our findings reaffirm the potential involvement of the peripheral serotoninergic system in cardiac remodeling and valve dysfunction in the context of AS. The SERAOPI pilot study [11] was the initial investigation that unveiled the aforementioned association. Despite its comprehensive examination through laboratory measurements, the need for further validation of the results raised from the relatively small and confined to a single-center sample size (n = 30). Notably, the control group comprised only 15 control volunteers who were significantly younger and lacked the comorbidities typically observed in elderly patients with severe AS, introducing a potential source of bias in the findings. In contrast, the control group in the present study was selected to match the AS patients in terms of gender, age, and comorbidities, with the exclusion of any form of AS. This ensured homogeneity between the two groups, a critical consideration given the documented role of serotonin in other cardiovascular conditions, such as heart failure [12,13], ischemic coronary artery disease [14], and arterial hypertension [15]. In addition, compared to the pilot study, which measured these biological parameters through aortic blood sampling, the current study aimed for a more clinically practical approach by conducting measurements from peripheral venous blood. However, after addressing these potential limitations and refining the methodology, our study achieved outcomes that not only reinforce the validity and clinical relevance of the conclusions drawn, but also emphasize that elevated levels of 5-HT and 5-HIAA may still represent a higher risk in patients with severe AS compared to both those without comorbidities and those with comorbidities but no AS.

Regarding laboratory parameters, the NT-proBNP levels were significantly higher in patients with aortic stenosis. Although the groups are homogeneous in terms of heart failure presence, the observed difference in NT-proBNP levels can be attributed to the under-lying pathology distinguishing them, namely severe aortic stenosis, which leads to increased intracardiac pressure. Correlations between NT-proBNP and serotonin or its metabolite were weak, suggesting that serotonergic activation in severe AS may not be directly related to neurohumoral activation.

In terms of echocardiographic parameters, interventricular septal and posterior wall thickness were greater in the AS group, reflecting left ventricular remodeling under chronic pressure overload. Similarly to laboratory findings, correlations with serotonin and its metabolite were weak, indicating that the observed ventricular hypertrophy is unlikely to be driven by serotonergic activity. Although serotonin plays a part in pulmonary vascular remodeling [16] and was shown to play a role in the development of adult pulmonary hypertension [17], we were unable to establish a correlation between increased plasma serotonin levels and pulmonary hypertension through echo-cardiographic marker PSAP (r = −0.017). However, the small size of the sample and the inability to measure pulmonary vascular resistance limit the ability to make conclusive inferences about the relationship between plasma serotonin levels and pulmonary hypertension in this case.

Serotonin’s influence on aortic stenosis is multifaceted, involving cellular proliferation, fibrosis, and calcification processes that contribute to the structural deterioration of the aortic valve. Experimental data indicate that serotonin can promote fibroblast proliferation, extracellular matrix deposition, and valvular interstitial cell activation, primarily through 5-HT_2_B receptor-mediated signaling. The activation of this pathway has been linked to enhanced expression of profibrotic mediators and osteogenic markers, providing a mechanistic basis for serotonin-induced valvular fibrosis and calcification [18,19]. Bouchareb and colleagues, through immune-gold scanning electron microscopy, detected platelet aggregates on diseased valves in both humans and mice and demonstrated that the release of platelet granules contributes to calcific aortic valve stenosis in an inflammatory mouse model [20]. A recent study found that 5-HT augmented TNF-α induction of matrix metalloproteinase-3 expression and induced osteoblastic differentiation and matrix mineralization of VIC [21]. On a more clinical and practical level, we conducted a novel investigation to assess the potential relationship between serotonin levels and the extent of valvular calcium, as measured by the computed tomography aortic valve calcium (CT-AVC) score, but the correlation was not confirmed. A hypothesis that warrants exploration in future studies is the use of a more sensitive approach, employing the aortic valve fibrosis score rather than the aortic valve calcium score, and incorporating all stages of aortic stenosis rather than limiting to severe cases.

In addition to AV changes, we report a weak association between elevated plasma serotonin and left ventricular hypertrophy. Animal models indicate that serotonin con-tributes to cardiac hypertrophy, primarily through serotonin 2B receptor activation and enhancement of angiotensin-II activity [22]. A recent study also showed an association between the high-serotonin group and higher odds of LVH on echocardiography [23]. Also consistent with our findings, their serotonin group was not significantly associated with decreased left ventricular ejection fraction [23].

In this study, 5-HIAA demonstrated a stronger discriminatory capability than 5-HT for predicting severe AS, as indicated by its higher AUC (0.704 vs. 0.641). Both biomarkers achieved statistical significance (*p* = 0.001 and *p* = 0.026, respectively), with 5-HIAA’s demonstrating particularly high sensitivity (86.8%). However, its relatively low specificity (47.4%) restricts its standalone diagnostic utility. In contrast, 5-HT exhibited moderate sensitivity (73.7%) but comparable specificity (50%), indicating that while it may not offer an advantage in diagnostic accuracy, it still holds relevance in clinical evaluation. 5-HIAA may outperform 5-HT in predicting severe AS because it reflects cumulative serotonergic activity through its role as the primary metabolite of serotonin, is more metabolically stable due to slower clearance, and integrates systemic and local serotonergic processes, unlike 5-HT levels that can fluctuate more dynamically due to rapid uptake and release by platelets or other cells.

This study presented a novel approach by investigating the role of serotonin as a potential key contributor to aortic valve stenosis, alongside its metabolite 5-hydroxyindoleacetic acid. In contrast to the previous pilot study, our research uniquely examined these molecules using peripheral venous samples, providing a minimally invasive method to evaluate their potential as biomarkers. By addressing the interplay between serotonin and 5-HIAA in this pathological context, this study contributes to the understanding of the serotoninergic system on disease mechanisms and highlights its prospective clinical utility in diagnosing or monitoring aortic valve stenosis.

### Limitations

Our study has certain limitations that warrant consideration. The most important limitation is the relatively small number of patients included, which restricts the statis-tical power and generalizability of the findings. Patient recruitment was also limited to two centers.

It should be acknowledged that, although other standard echocardiographic parameters for assessing aortic stenosis severity were measured (mean gradient, aortic valve area, and Doppler velocity index), only the peak transvalvular velocity (Vmax) was included in the intergroup comparison to maintain consistency in data presentation. Furthermore, stroke volume and left atrial volume were not consistently available, representing a methodological limitation of the echocardiographic assessment.

In addition, serotonin levels were measured from stored serum samples, which may have introduced variability or affected the accuracy of the results compared to fresh samples.

These limitations underscore the importance of conducting larger, multicenter studies with standardized methodologies, including the use of other objective measures such as the aortic valve fibrosis score.

## 4. Materials and Methods

### 4.1. Study Design and Patient Selection

This cross-sectional, case–control study enrolled 76 patients referred to the Cardiology Departments of the “Niculae Stăncioiu” Heart Institute and the Clinical Rehabilitation Hospital in Cluj-Napoca, Romania, spanning the period from April 2022 to August 2023. The case cohort comprised 38 patients diagnosed with severe calcific aortic stenosis (AS) based on established echocardiographic criteria. In parallel, 38 control patients were selected during the same period and frequency-matched to cases for age, sex, and major comorbidities (excluding AS of any severity) to ensure comparable group distributions. Exclusion criteria in both groups encompassed other significant valvular heart disease, carcinoid tumors, and depression, as well as the usage of medications or consumption of foods that could potentially influence the levels of serotonin (5-HT) and 5-hydroxyindoleacetic acid (5-HIAA).

### 4.2. Ethics

The research protocol received approval from the Ethics Committee of the “Iuliu Haţieganu” University of Medicine and Pharmacy in Cluj-Napoca, Romania (approval no. 76/14 March 2022), as well as from the Ethics Committee of the Heart Institute in Cluj-Napoca (approval no. 4540/12 April 2022). The study was carried out in accordance with the principles of the Declaration of Helsinki, and written informed consent was obtained from all participating patients.

### 4.3. 5-HT and 5-HIAA Measurements

All patients had fasting blood samples collected in additive-free vacutainers, which were transported and stored at −80 degrees Celsius at the Research Center for Functional Genomics, Biomedicine and Translational Medicine at the University of Medicine and Pharmacy Iuliu Haţieganu until the formation of the total batch, when serum 5-HT and 5-HIAA levels were determined by enzyme-linked immunoassay (ELISA).

Serum levels of serotonin (5-HT) and its metabolite 5-hydroxyindoleacetic acid (5-HIAA) were quantified using commercially available sandwich ELISA kits: 5-HT (Elabscience Biotechnology Inc., Houston, TX, USA; Catalog No. E-EL-003) and 5-HIAA (FineTest, Wuhan Fine Biotech Co., Ltd., Wuhan, China, Catalog No. EU2582). ELISAs were performed using a Biotek Synergy H1 Hybrid absorbance microplate reader (BioTek Instruments, Inc., Winooski, VT, USA) according to the manufacturers’ instructions. Absorbance values from each serum sample were plotted against the standard curve provided with each kit, and concentrations were extrapolated using the corresponding representative model in GraphPad Prism 6 software. Detailed information on calibration curves, detection limits, and intra- and inter-assay variability are provided by the manufacturers.

### 4.4. Statistical Analysis

The statistical analysis was performed using MedCalc Statistical Software version 22.016 (MedCalc Software Ltd., Ostend, Belgium; http://www.medcalc.org, accessed on 7 January 2024). Normality assessment was conducted through the Kolmogorov–Smirnov test. Continuous variables with a normal distribution were expressed as mean ± standard deviation (SD) and compared using the Student’s *t*-test. For variables not normally distributed, data were presented as medians with interquartile ranges (Q1–Q3) and analyzed using the Mann–Whitney U test. Categorical variables were described as frequencies and percentages, and differences between groups were evaluated with the chi-square test. Patients were stratified according to the presence or absence of severe aortic stenosis, forming the case and control groups, respectively. Correlations among parameters within the AS group were examined using Spearman’s rank correlation coefficients.

Receiver operating characteristic (ROC) curve analysis was performed with a bootstrap-based Youden index confidence interval to determine the optimal threshold and its 95% CI. This approach was used to identify the cut-off values of 5-HT and 5-HIAA that best predicted severe aortic stenosis by maximizing both sensitivity and specificity. With 38 patients per group, the study is powered to accurately estimate the AUC for both 5-HT and 5-HIAA, with expected values ranging between 0.6 and 0.8. This sample size provides adequate precision to assess the diagnostic potential of these biomarkers in distinguishing aortic stenosis patients from controls. Statistical significance was defined as a *p*-value less than 0.05.

## 5. Conclusions

Our study supports previous observations of altered serum levels of 5-HT and its MAO-A-dependent metabolite, HIAA, in patients with severe aortic stenosis. These findings further suggest a possible involvement of serotonergic activation in the pathophysiology of the disease. Nevertheless, given the cross-sectional, observational design and the limited sample size, causality cannot be inferred. Future larger and prospective studies are needed to confirm these results and to better understand the potential role of the serotonergic system in the development and progression of aortic stenosis.

## Figures and Tables

**Table 1:** Demographic characteristics, comorbid conditions, cardiovascular medication, and echocardiographic measurements of the patients according to the presence of AS.

Population Characteristics		Total Patients n = 76	Severe AS n = 38	Control n = 38	*p*-Value
Age (years)		76.5 ± 5	76.6 ± 5.7	76.5 ± 4.4	0.893
Gender (male)		37 (48.7%)	15 (39.4%)	22 (57.8%)	0.110
Body mass index (kg/m^2^)		27.9 (24.8–31.2)	26.9 (24.9–31.2)	28.5 (24.5–31.2)	0.677
Current smoking		6 (7.9%)	4 (0.1%)	2 (0.05%)	0.398
**Comorbidities**					
	Arterial Hypertension	69 (90.7%)	34 (89.4%)	35 (92.1%)	0.691
	Hyperlipidemia	60 (78.9%)	29 (76.3%)	31 (81.5%)	0.576
	Diabetes mellitus	24 (31.6%)	13 (34.2%)	11 (28.9%)	0.623
	Coronary artery disease	36 (47.3%)	14 (36.8%)	22 (57.8%)	0.066
	History of myocardial revascularization	16 (21.1%)	7 (18.4%)	9 (23.6%)	0.576
	Atrial fibrillation	26 (34%)	8 (21%)	18 (47%)	**0.015**
	Heart failure (NYHA class II-IV)	71 (93.4%)	37 (97.3%)	34 (89.4%)	0.167
**Cardiovascular medication**					
	CCB	31 (40.8%)	13 (34.2%)	18 (47.3%)	0.246
	ARBs/ACEi	60 (78.9%)	24 (63.1%)	36 (94.7%)	**0.0008**
	BB	57 (75%)	27 (71%)	30 (78.9%)	0.429
	MRA	24 (31.6%)	14 (36.8%)	10 (26.3%)	0.326
	Statins	58 (76.3%)	30 (78.9%)	28 (73.6%)	0.591
**Echocardiography parameters**					
	Aortic valve Vmax (m/s)	2.9 (1.5–4.4)	4.4 (4.2–4.9)	1.5 (1.3–1.7)	**<0.0001**
	Ascending aorta (mm)	34.4 ± 4	34.1 ± 4.6	34.7 ± 3.3	0.477
	LA (mm)	45 (41.5–51)	46.5 (43–51)	45 (41–50)	0.368
	LVEDD (mm)	49.2 ± 6.1	48.5 ± 6.8	50 ± 5.3	0.315
	LVESD (mm)	32.5 (28–39)	32 (28–39)	34.5 (29–39)	0.727
	IVS (mm)	13.6 ± 2.8	15.5 ± 2.2	11.6 ± 1.9	**<0.0001**
	LVPW (mm)	12.1 ± 2.4	13.4 ± 2.1	10.7 ± 1.9	**<0.0001**
	E/e’	12.5 ± 4.4	14.8 ± 3.4	10.2 ± 4.1	**<0.0001**
	PSAP (mmHg)	35 (30–46)	40 (33–49)	35 (29–43)	0.067
	LVEF (%)	51 (45–58)	50.5 (45–55)	53 (45–60)	0.271

Data are presented as mean ± standard deviation (SD), median and quartiles Q1 and Q3 or n (%). ARBs: Angiotensin receptor blockers; ACEi: Angiotensin-converting enzyme inhibitors; BB: beta blockers; CCB: Calcium channel blockers; IVS: Interventricular septum; LA: Left atrium; LVEDD: Left ventricular end diastolic diameter; LVEF: Left ventricular ejection fraction; LVPW: Left ventricular posterior wall; LVESD: Left ventricular end systolic diameter; MRA: Mineralocorticoid receptor antagonist; NYHA: New York Heart Association; PSAP: Pulmonary systolic arterial pressure. Bold emphasizes the statistically significant results.

**Table 2:** Laboratory parameters of the patients according to the presence of AS.

Biochemical Markers	Total Patients n = 76	Severe AS n = 38	Control n = 38	*p*-Value
Hemoglobin (g/dL)	13 ± 1.8	13 ± 1.8	12.9 ± 1.8	0.750
Creatinine (mg/dL)	1 (0.8–1.2)	0.9 (0.7–1.2)	1 (0.8–1.3)	0.167
NT-proBNP (pg/mL)	1582.5 (871.5–2801)	2536 (1221–4128)	1006.5 (540–1956)	**0.0001**
Total cholesterol (mg/dL)	150 (129.3–182)	144.7 (127.5–182.1)	163 (130–182)	0.248
LDL-C (mg/dL)	90.5 ± 34.9	83.8 ± 33.7	97.2 ± 35.2	0.095
HDL-C (mg/dL)	44.8 (37–56.8)	46 (37.9–58)	43.5 (37–53)	0.529
Triglycerides (mg/dL)	98.7 (83.2–134.5)	95.1 (72–113.4)	112 (85–173)	0.181
Glucose (mg/dL)	103 (95–116.5)	103.5 (98–117)	103 (95–116)	0.429
5-HT (ng/mL)	1036.4 (903.4–1106.6)	1066.5 (961.9–1112)	977.4 (394.3–1097.9)	**0.034**
5-HIAA (ng/mL)	52.3 ± 14.7	57 ± 12.7	47.5 ± 15.3	**0.004**

Data are presented as mean ± standard deviation (SD), median and quartiles Q1 and Q3 or n (%). HDL-C: High-density lipoprotein cholesterol; 5-HT: 5-Hydroxytryptamine; 5-HIAA: 5-Hydroxyindolacetic acid. Bold emphasizes the statistically significant results.

**Table 3:** Correlation analysis between the two biomarkers and clinical parameters in severe AS.

	Vmax	Mean PG	LVPW	IVS	E/e’	NT-proBNP	Age
5-HT	0.23	0.2	0.22	0.08	0.16	0.27	−0.31
5-HIAA	0.26	−0.01	0.2	0.31	0.22	0.2	−0.01

Data are presented as correlation coefficients (r) between serotonin (5-HT) and its metabolite (5-HIAA) with various echocardiographic and clinical parameters in patients with severe aortic stenosis. Correlations are categorized as weak positive correlation (r = 0.2–0.4) or negligible (r < 0.2). 5-HT: 5-Hydroxytryptamine; 5-HIAA: 5-Hydroxy indolacetic acid; IVS: Interventricular septum; LVPW: Left ventricular posterior wall; Mean PG: aortic valve mean pressure gradient; Vmax: aortic valve maximum velocity.

## Data Availability

The original contributions presented in this study are included in the article. Further inquiries can be directed to the corresponding author.
